# Clinicopathological Analysis of Prostate Cancer in Young Men: Experience of a Tertiary Referral Hospital in Western Romania

**DOI:** 10.3390/medicina62081465

**Published:** 2026-07-28

**Authors:** Vlad Dema, Alexei Croitor, Robert A. Barna, Sorin Dema, Sorina Tăban, Mihail Nanu, Daiana Mîrnea, Victor Cădariu-Brăiloiu, Răzvan Bardan, Alin A. Cumpănaș

**Affiliations:** 1 Department XV, Discipline of Urology, “Victor Babes” University of Medicine and Pharmacy, 300041 Timisoara, Romania; vlad.dema@umft.ro (V.D.); razvan.bardan@umft.ro (R.B.); cumpanas.alin@umft.ro (A.A.C.); 2 Department of Urology, “Pius Brinzeu” Emergency County Hospital, 300723 Timisoara, Romania; victor.cadariu-brailoiu@rezident.umft.ro; 3 Doctoral School, “Victor Babes” University of Medicine and Pharmacy, 300041 Timisoara, Romania; robert.barna@umft.ro; 4 Anapatmol Research Center, “Victor Babes” University of Medicine and Pharmacy, 300041 Timisoara, Romania; taban.sorina@umft.ro (S.T.); mihail.nanu@rezident.umft.ro (M.N.); daianaalinamirnea@gmail.com (D.M.); 5 Department of Pathology, “Pius Brinzeu” Emergency County Hospital, 300723 Timisoara, Romania; 6 Department of Oncology, “Victor Babes” University of Medicine and Pharmacy, 300041 Timisoara, Romania; sorin.dema@umft.ro

**Keywords:** prostate cancer, young patients, serum PSA, Gleason score, grade group, intraductal carcinoma, western Romania, risk stratification

## Abstract

*Background and Objectives*: Prostate cancer (PC) has traditionally been regarded as a disease of older men, most commonly diagnosed after the age of 70 years. Recent epidemiological data indicate an increasing incidence of this malignancy among men younger than 55 years, with important clinical and therapeutic implications. The present study aimed to analyze the epidemiological, clinical, and pathological characteristics of PC in young patients diagnosed at a tertiary referral urology center in western Romania. *Materials and Methods*: Medical records and the electronic database of the “Pius Brînzeu” County Emergency Clinical Hospital in Timișoara (PBCECHT) were reviewed to identify men aged ≤55 years diagnosed with PC over a 10-year period (2015–2024). Collected data included: the age at diagnosis, serum prostate-specific antigen (PSA) levels, specimen type, histologic type, Gleason score/International Society of Urological Pathology (ISUP) grade group, tumor stage, and additional prognostic factors. *Results*: Of the 1742 patients diagnosed with PC during the study period, 70 (4.02%) were aged ≤55 years. The average age at diagnosis for the group of young men was 52.47 years. Most patients were from urban areas (64.29%). The PSA was available in 51/70 cases (range: 1.35–5182; median: 15; IQR: 51). The mean Gleason score was 7.15, and the mean ISUP grade group was 2.5. Histologically, acinar adenocarcinoma predominated, while a minority of cases showed mixed acinar and ductal/adenocarcinomas with ductal features. Most tumors (70%) were clinically significant. *Conclusions*: Although less common, PC in young men often exhibits aggressive features. It is crucial to consider genetic risk factors and a multidisciplinary approach for optimal management.

## 1. Introduction

Prostate cancer (PC) has traditionally been associated with advanced age, with most cases diagnosed in patients older than 70 years [1]. However, more than 10% of all PC cases identified in the United States have been reported in patients younger than 55 years [2], and less than 1% in individuals younger than 44 years [3]. Autopsy studies suggest a substantially higher prevalence of PC among younger men, estimated at 20–30% or even higher [4].

The natural history, biology, and management of these tumors are important because, according to several studies, their incidence/prevalence is increasing [5], while the diagnosis of this malignancy in this segment of the male population and the clinical and therapeutic implications of a younger age at diagnosis have caused a lot of concern.

The present study aimed to analyze PC in young patients presenting to a tertiary referral urology center in western Romania, in order to delineate the epidemiological–demographic, clinical, and pathological profile of this malignancy and, based on the results obtained, to formulate recommendations that may contribute to optimizing the diagnosis and/or surveillance of young patients with PC.

## 2. Materials and Methods

### 2.1. Study Design

This was a retrospective observational study based exclusively on the analysis of pre-existing clinical and pathological data, without any direct intervention involving patients.

### 2.2. Selection Criteria

Inclusion criteria:

− patients with PC;
− age ≤ 55 years at the time of diagnosis;
− histopathological diagnosis was established at the “Pius Brînzeu” County Emergency Clinical Hospital in Timișoara (PBCECHT) between 1 January 2015 and 31 December 2024.

Exclusion criteria:

− prostatic involvement by other tumor types (urothelial carcinomas, sarcomas, lymphomas, etc.).

We selected 55 years as the age cut-off because this threshold is the most commonly used definition of early-onset prostate cancer in the published literature, particularly in epidemiological and germline genetic studies [6,7,8].

### 2.3. Data Collection

Data were extracted from our hospital’s electronic database and clinical records using the following keywords: “prostate cancer”, “prostatic carcinoma”, “carcinoma of the prostate”, and “prostatic adenocarcinoma”. In addition to patients admitted to or treated in the Department of Urology, patients with PC diagnosed outside PBCECHT who showed up at the hospital’s Department of Pathology for histopathologic diagnosis were also included.

For patients with two entries in the database (initial diagnosis upon prostate needle biopsy [CNB] specimen and subsequent diagnosis upon radical prostatectomy [RP] specimen), only the data from the diagnostic biopsy were recorded.

The following information was collected:

− age at diagnosis;
− residence (urban/rural);
− pre-biopsy/pre-intervention serum PSA level;
− specimen type on which the diagnosis of PC was established;
− histological tumor type;
− Gleason score and WHO/ISUP grade group;
− pathologic stage and surgical margin status (R status) for RP-treated cases;
− additional prognostic factors: intraductal carcinoma of the prostate (IDC-P), neuroendocrine (NE) differentiation, perineural invasion (PNI), and lymphovascular invasion (LVI).

Grading and tumor staging were performed according to the WHO and TNM/AJCC criteria in force at the time of diagnosis [9,10,11].

For tumors showing two distinct tumor populations with different Gleason scores (for example, acinar and ductal adenocarcinoma), the higher Gleason score and grade group were retained for the respective case.

Patients diagnosed by core needle biopsy (CNB) specimens were assigned to risk groups according to ESMO recommendations [12], as follows:

• low risk: T1–T2a, Gleason score ≤ 6, PSA ≤ 10 ng/mL;
• intermediate risk: T2b, Gleason score 7, and/or PSA 10–20 ng/mL;
• high risk: ≥T2c, Gleason score 8–10, or PSA > 20 ng/mL.

Tumors were considered clinically significant if they displayed at least one of the following features: stage ≥ pT3, nodal metastasis, Gleason score ≥ 7 (or Gleason pattern 4/5), positive surgical margins, or metastatic disease [13,14,15,16].

To compare the data of the young PC patient cohort with those of all patients diagnosed with PC at PBCECHT, we used data regarding the main characteristics of all patients diagnosed with PC in the same institution between 1 January 2015 and 31 December 2024.

For some of the cases, immunohistochemistry (IHC) analysis was performed at the time of diagnosis in order to document IDC-P, NE differentiation, and the prostatic origin of metastases.

For the IHC investigation, prediluted monoclonal antibodies against p63, CK5, PSA, NKX3.1, CK7, CK20, Synaptophysin, Chromogranin A, TTF1, and CDX2 were used on the automated Bond Max platform (Leica Biosystem, Deer Park, IL, USA) according to the manufacturer’s instructions.

### 2.4. Statistical Analysis

Data management, calculations, and graphical representations were performed in Microsoft Excel (Microsoft Corporation, Redmond, WA, USA) and GraphPad Prism version 9.3.0 (GraphPad Software, San Diego, CA, USA). Because PSA values were markedly skewed, they are reported as the median and interquartile range and were log-transformed [ln(PSA + 1)] for statistical comparisons. Differences in PSA and grade group between groups were assessed using the Mann–Whitney U test and chi-square test, respectively, with effect sizes and 95% confidence intervals reported alongside *p*-values. Logistic regression was used to identify factors associated with clinically significant and high-grade disease, and to assess trends over the study period, with results expressed as odds ratios and 95% confidence intervals. A *p*-value < 0.05 was considered statistically significant. Histological sections were examined, and photomicrographs were acquired using an Olympus BX43 microscope with an Olympus DP74 digital camera and Olympus cellSens Dimension software version 2.3 (Olympus Corporation, Tokyo, Japan).

## 3. Results

### 3.1. Epidemiological and Demographic Data on PC in Young Men

Over the 10-year period (2015–2024), 1742 patients with PC were diagnosed in the Department of Pathology of the PBCECHT.

During the same period, 76 patients aged ≤55 years with carcinoma of the prostate (primary or metastatic) were identified. After exclusion of ineligible cases (according to the study inclusion/exclusion criteria), the final study cohort comprised 70 cases of PC in young patients, representing 4.02% of all PC patients diagnosed over the 10-year study period.

Table 1 presents the main characteristics of patients older than 55 years, as well as those of the young patient subgroup (aged 45–55 years), summarizing statistics on age at diagnosis, PSA level, Gleason score, and grade group.

The main findings relating only to the young patient cohort are presented briefly in Table 2 and in more detail in each subsection of the Results.

### 3.2. Distribution of Young PC Cases According to Year of Diagnosis

**Figure 1:**
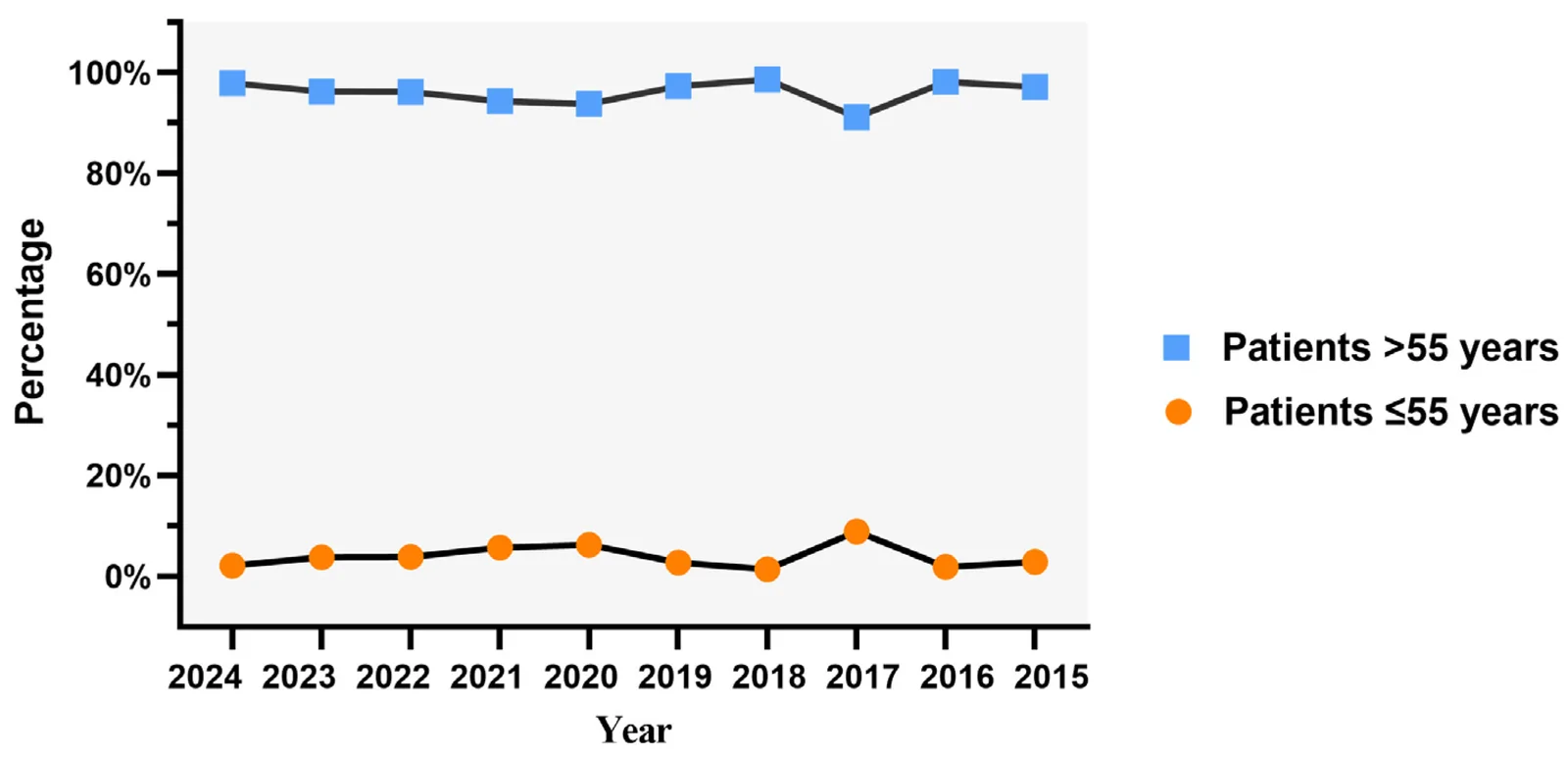
Percentage of young (≤55 years) and old (>55 years) PC patients diagnosed each year, 2015–2024.

Over the 10-year study period (2015–2024), 70 of 1742 patients (4.02%) diagnosed with PC were young (≤55 years). The annual proportion of young patients ranged from 1.40% (2018) to 8.89% (2017), with no consistent temporal pattern (Figure 1). The Cochran–Armitage test revealed no significant trend in the proportion of young patients over time (z = −0.36, *p* = 0.72), and logistic regression with calendar year as a continuous predictor confirmed this finding (OR = 0.99 per year, 95% CI 0.91–1.07, *p* = 0.72). These results indicate that the relative burden of prostate cancer among younger patients remained stable across the study period, with year-to-year variation consistent with random fluctuation.

### 3.3. Distribution of Cases by Age Group and Area of Residence

**Figure 2:**
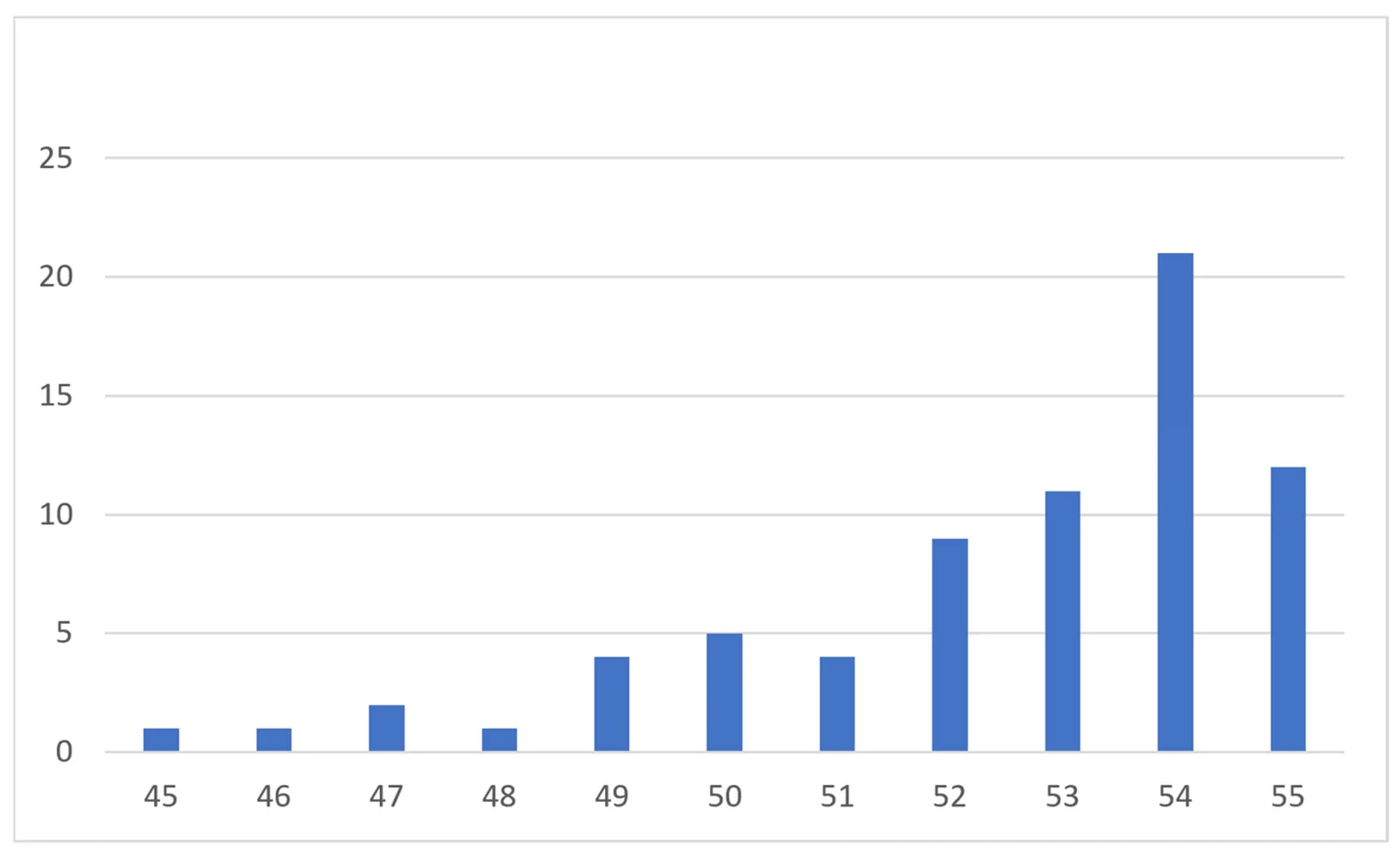
Distribution of cases according to age at diagnosis (n = 70).

All patients were between 45 and 55 years of age, with none younger than 45 years (Figure 2). A general upward trend in the number of cases was observed with increasing age, peaking at 54 years. Most patients, 45/70 (64.29%), were from urban areas.

### 3.4. Serum PSA Values

Serum PSA at diagnosis was available for 51 of 70 young patients. Given the markedly skewed distribution of values, the median and interquartile range are reported in Table 2, along with data from log transformation. Based on raw data, PSA levels were higher in young patients compared with the ones older than 55 years old, diagnosed during the study period, with a median of 15 ng/mL compared to 12 ng/mL, although this difference did not reach statistical significance, as shown by a Mann–Whitney U statistic of 35,798, *p* = 0.33 and a rank-biserial correlation of −0.08, indicating a negligible effect size. Following log transformation, the geometric mean ratio between the two groups was 1.44, with a 95% confidence interval ranging from 0.88 to 2.37. A Welch’s *t*-test performed on ln(PSA + 1) yielded a *p*-value of 0.146, further confirming the absence of a statistically significant difference between groups.

### 3.5. Types of Specimens Analyzed

**Figure 3:**
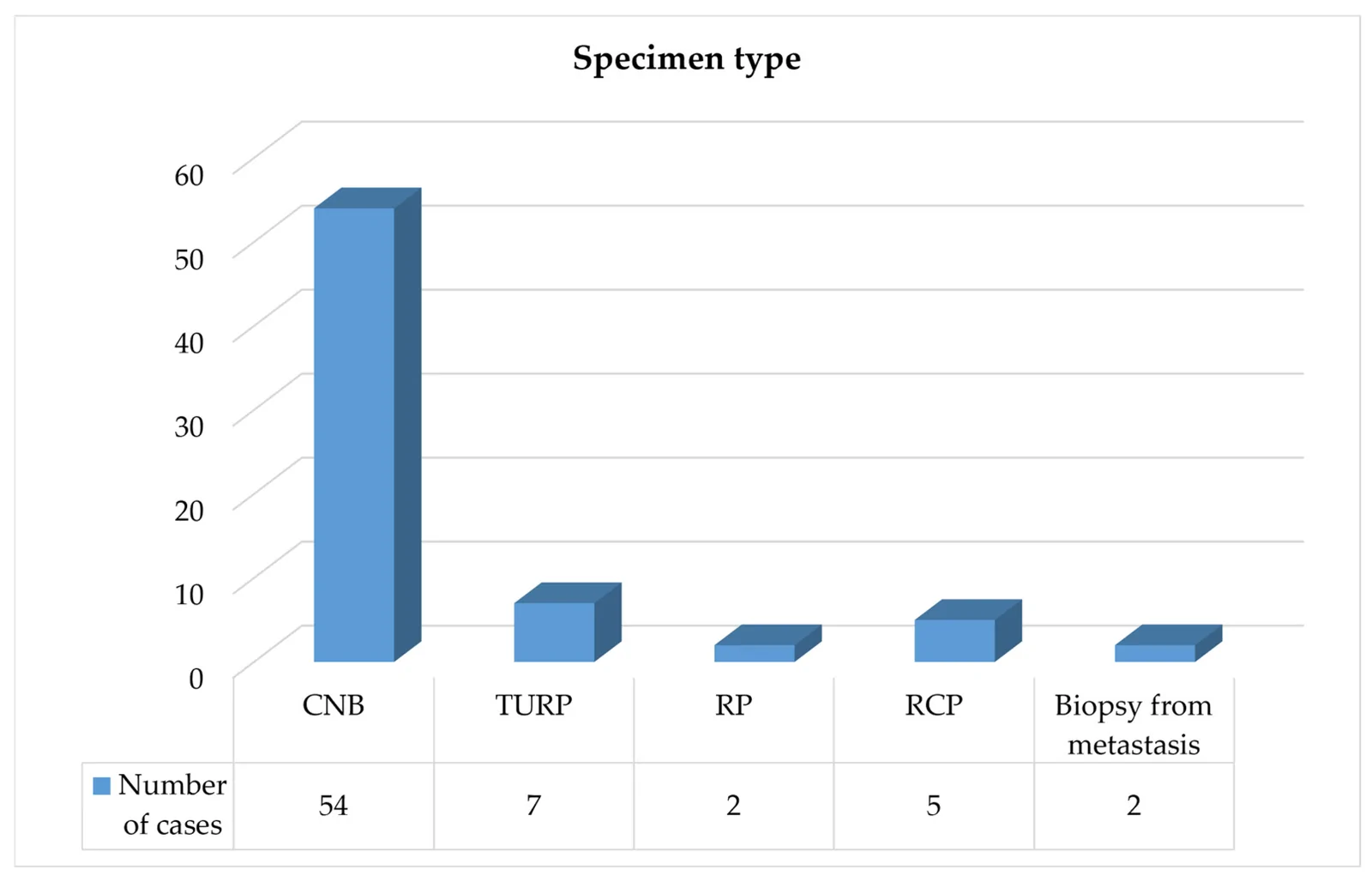
Distribution of cases by specimen type (n = 70).

Most cases of PC in young patients (54/70–77.14%) were diagnosed by CNB specimens (Figure 3).

In seven cases (10%), PC was diagnosed during examination of transurethral resection of the prostate (TURP) specimens performed in patients with lower urinary tract obstruction symptoms. These seven cases had the following Gleason scores: Gleason score 6 (3 + 3) in one case, Gleason score 7 (3 + 4) in one case, Gleason score 8 in three cases (4 + 4 in two cases; 3 + 5 in one case), and Gleason score 9 in two cases (4 + 5 in one case; 5 + 4 in one case). Regarding the prognostic grade group, they were classified as follows: grade group 1 in one case, grade group 2 in one case, grade group 4 in three cases, and grade group 5 in two cases.

Other specimen types in which PC was diagnosed included radical cystoprostatectomy (RCP) in five cases (7.14%) and radical prostatectomy (RP) in two cases (2.86%), in which the first presentation to our hospital was during RP, with the biopsy being examined in another laboratory. Five patients in the cohort diagnosed by CNB subsequently underwent RP at our institution, so the RP pathology results were available. Thus, the analyzed RP cohort comprised seven cases.

All PC cases identified in RCP specimens were organ-confined tumors (pT2N0) with Gleason score 6 and grade group 1, whereas PC cases identified in RP specimens (seven in total) were extraprostatic tumors—pT3a/b—in five cases (71.42%), with one with lymph node metastasis (pN1), and were confined to the prostate (pT2N0) in only two cases. Gleason score distribution in RP specimens was as follows: score 6 in one case, score 7 in five cases (3 + 4 in four cases and 4 + 3 in one case), and score 9 in one case, corresponding to grade groups 1 in one case, 2 in four cases, 3 in one case, and 5 in one case.

**Figure 4:**
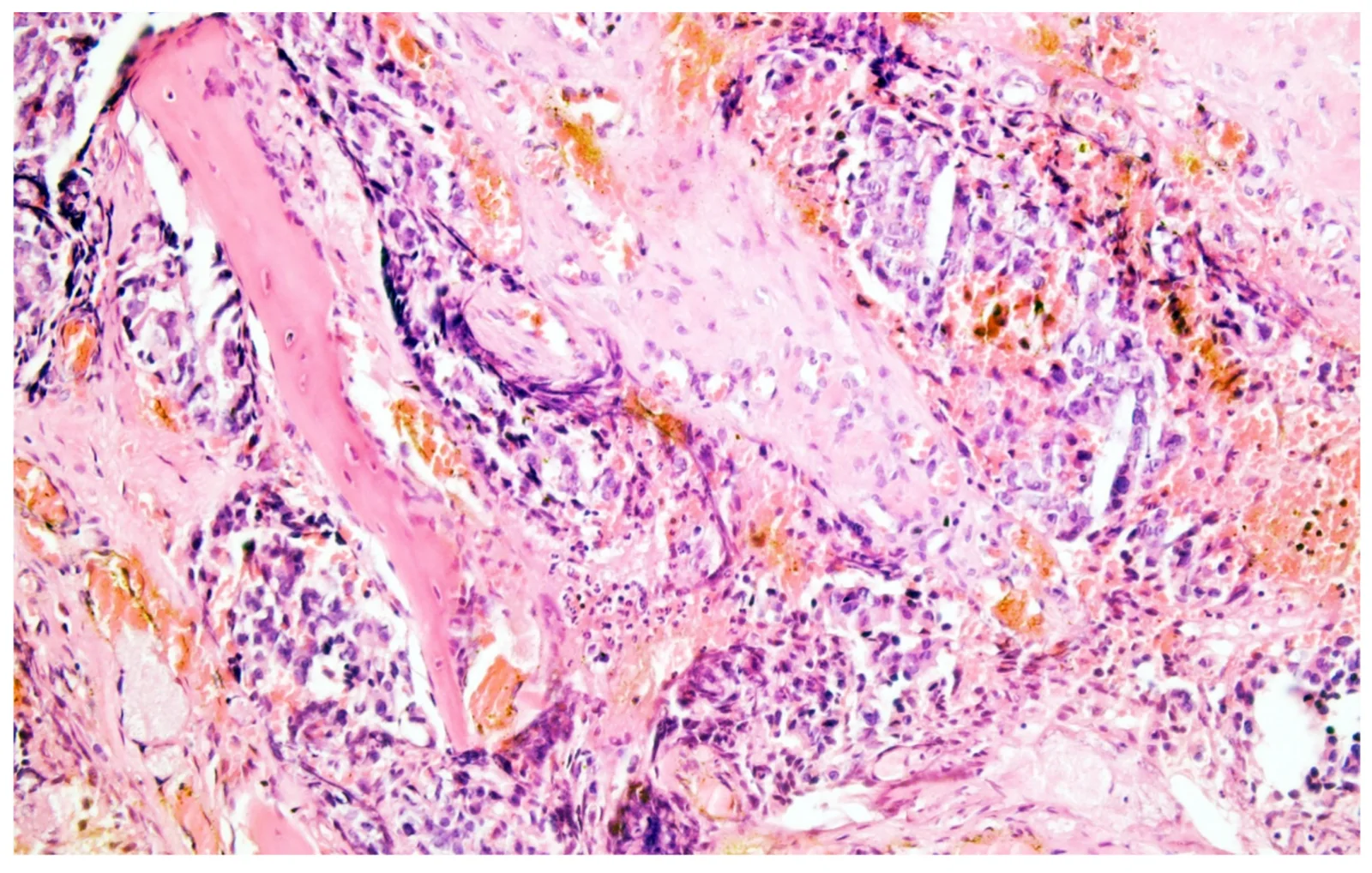
Tumor cells infiltrate between bony lamellae. HE, ×200.

**Figure 5:**
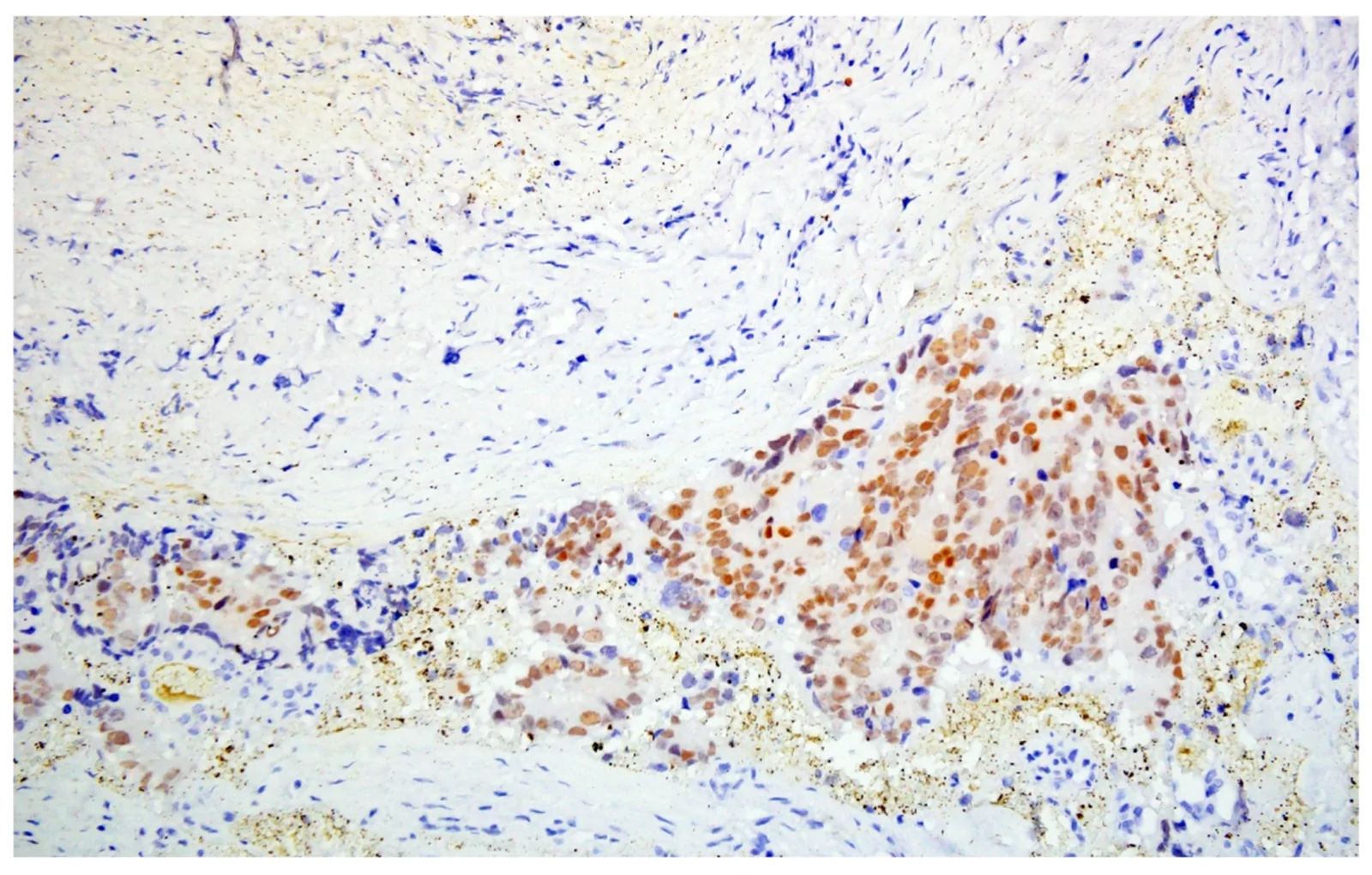
Positive reaction for NKX3.1 confirms prostatic origin of the tumor. Anti-NKX3.1, DAB, counterstain with hematoxylin, ×200.

In two cases (3% each), the diagnosis of PC was established upon microscopic examination of a biopsy from vertebral bone metastasis (Figure 4 and Figure 5) and brain metastasis, respectively.

### 3.6. Distribution of PC Cases Diagnosed by CNB Specimens, According to ESMO Risk Group

**Figure 6:**
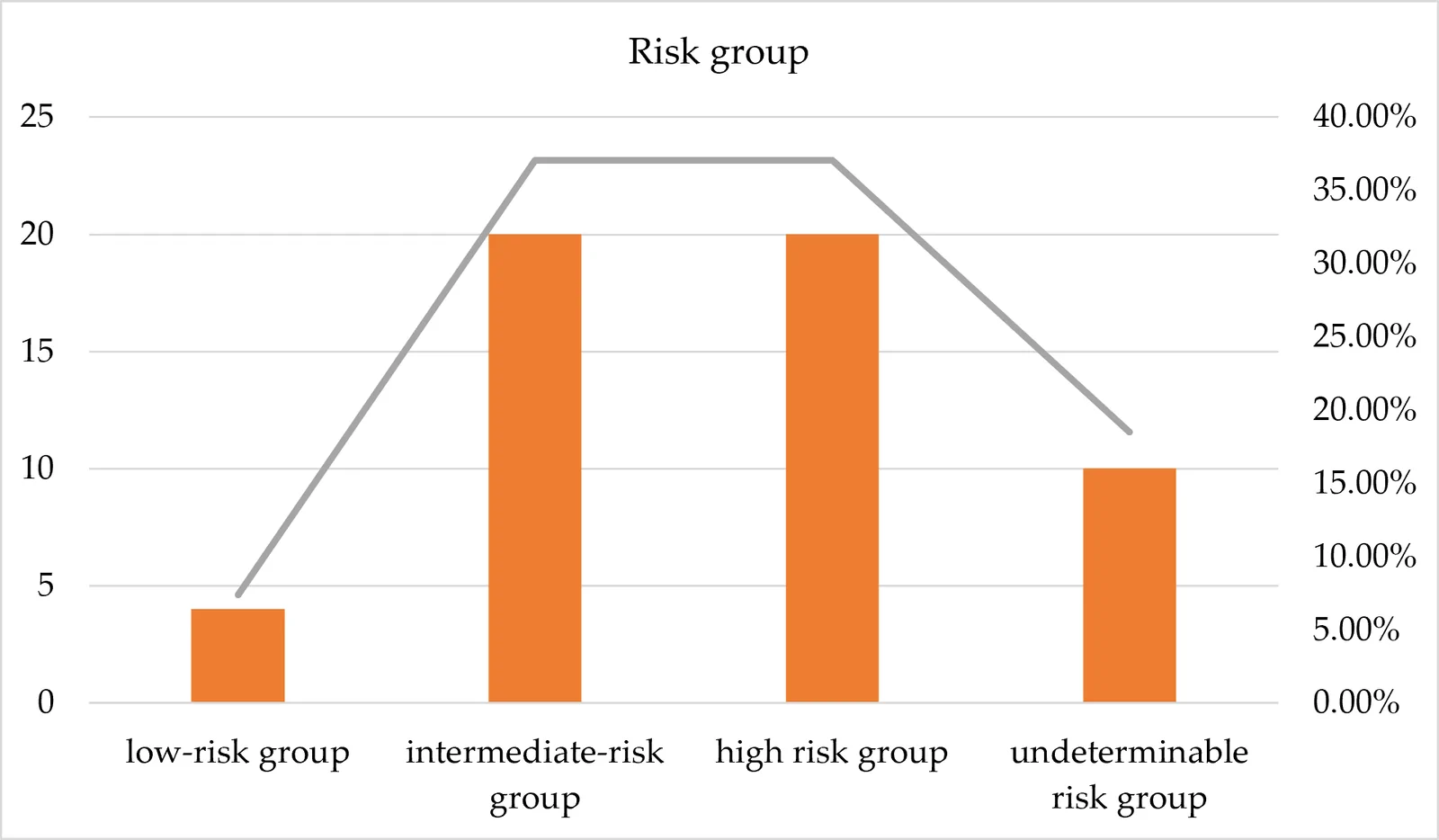
Distribution of PC cases diagnosed with CNB specimens according to risk group (n = 54).

Of the fifty-four patients diagnosed by CNB specimens, four (7.40%) cases belonged to the low-risk group, while twenty cases each (37.04%) were classified as intermediate-risk and high-risk, respectively (Figure 6). In 10 patients (18.52) for whom serum PSA values were unavailable, risk-group classification could not be performed.

### 3.7. Histological Type of the Tumors

**Figure 7:**
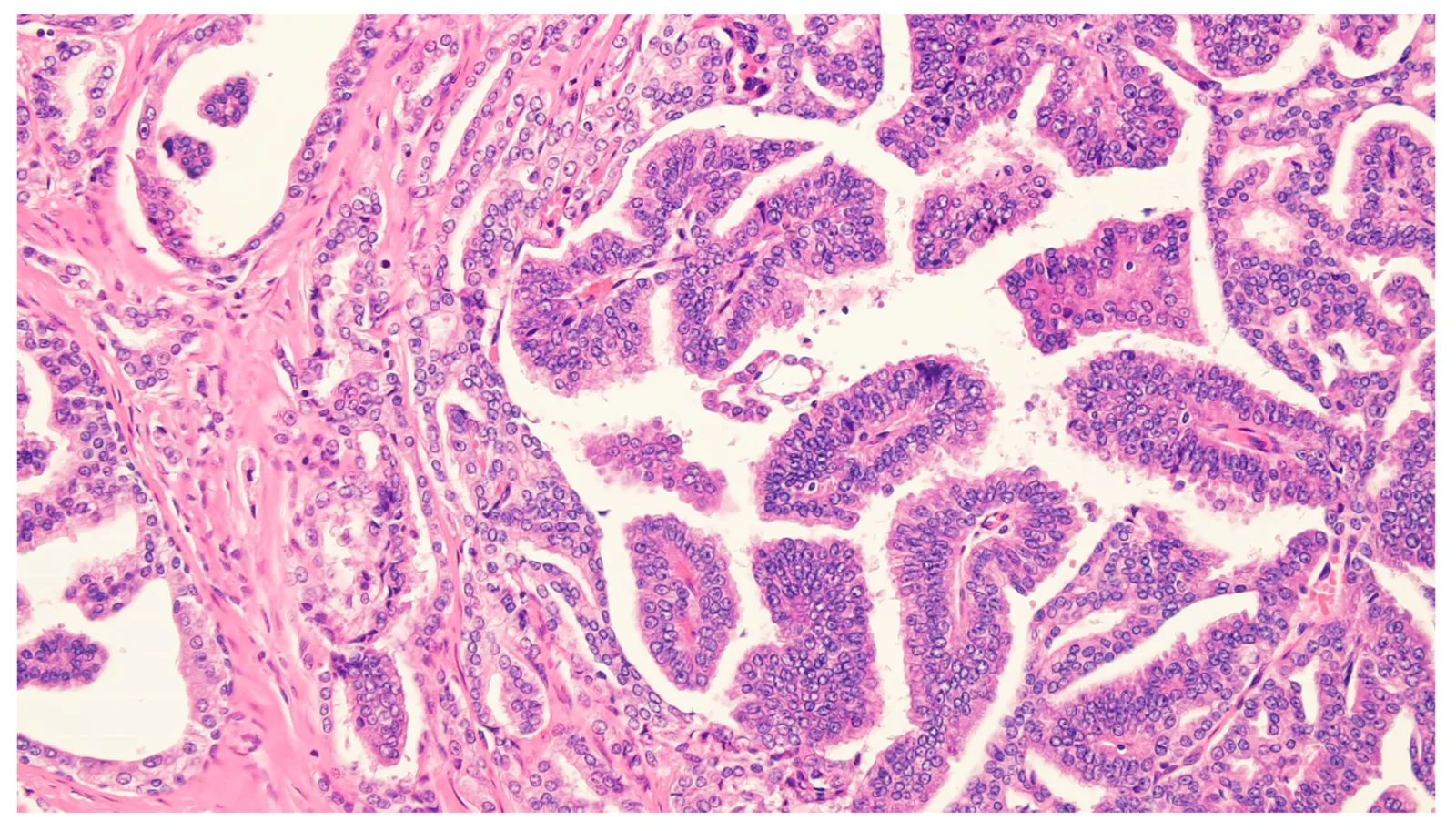
Mixed acinar and ductal carcinoma. HE, ×200.

**Figure 8:**
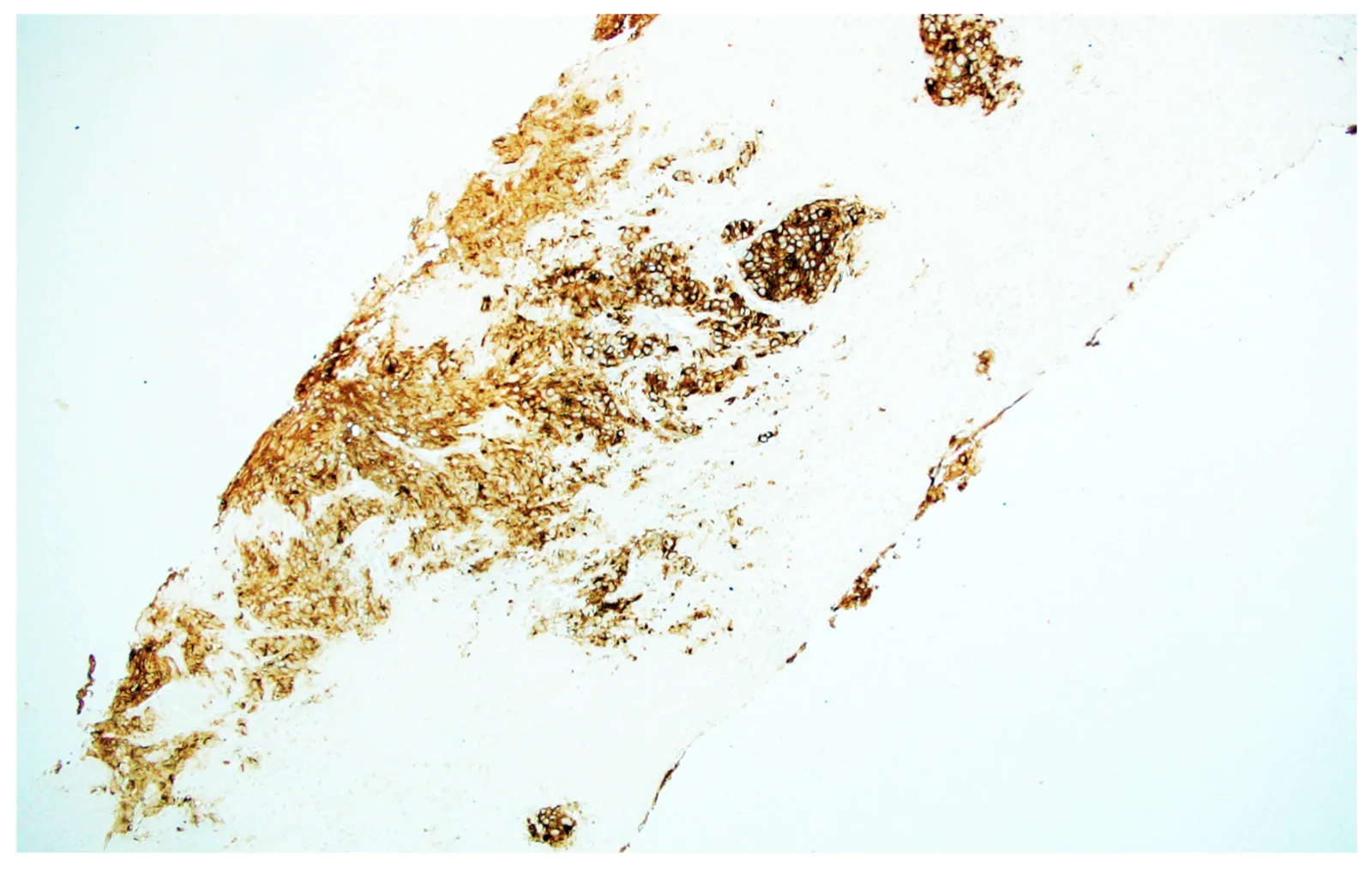
Extensive NE differentiation in a poorly differentiated adenocarcinoma. Anti-Syn, DAB, counterstain with hematoxylin, ×100.

**Figure 9:**
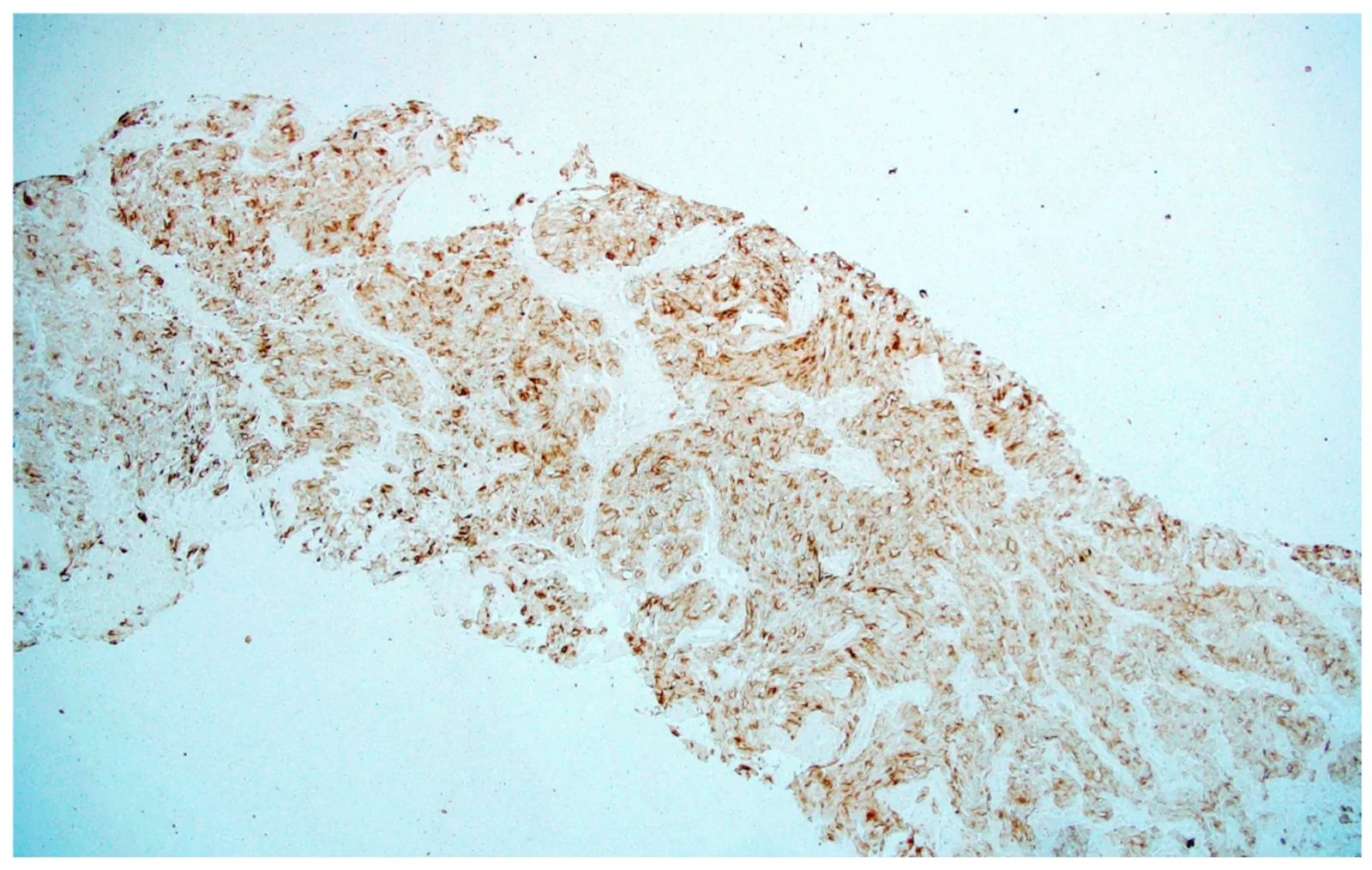
Positive reaction for PSA in the same case as in Figure 8. Anti-PSA, DAB, counterstain with hematoxylin, ×100.

**Figure 10:**
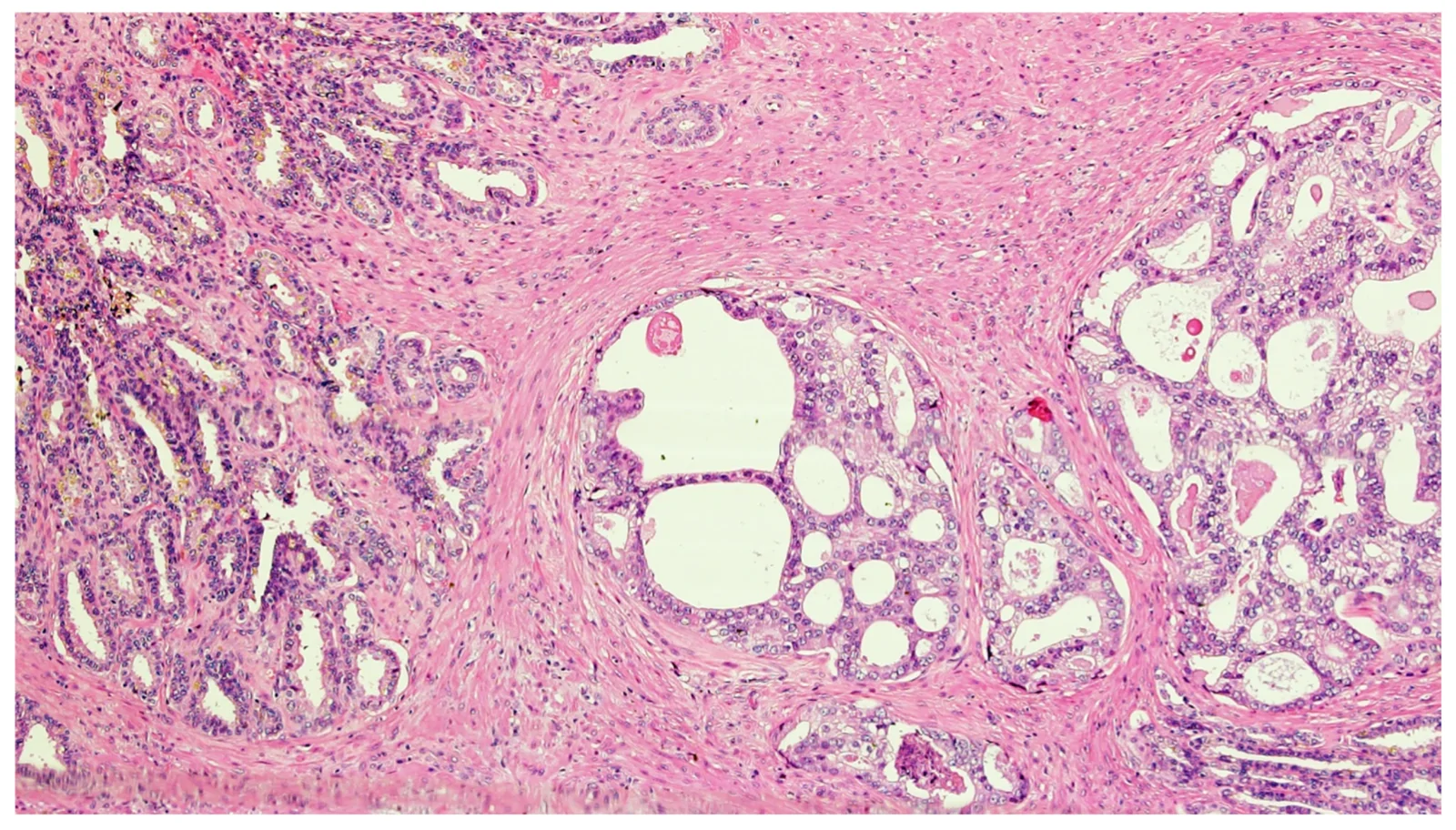
Cribriform adenocarcinoma invading seminal vesicles. HE, ×100.

Most tumors were acinar adenocarcinomas: sixty-eight cases (97.14%), while two cases (2.86%) were mixed adenocarcinomas (acinar and ductal/with ductal features) (Figure 7). Two of the seventy cases (2.86%) of poorly differentiated acinar adenocarcinomas (with Gleason scores of 9 and 10, respectively) showed patchy and diffuse NE differentiation, demonstrated by positive immunostaining for NE markers (chromogranin A ± synaptophysin) and PSA (Figure 8 and Figure 9). Invasive cribriform carcinoma was present in 24 cases (34.28%) (Figure 10).

### 3.8. Associated Microscopic Findings, Gleason Score, and Grade Group

**Figure 11:**
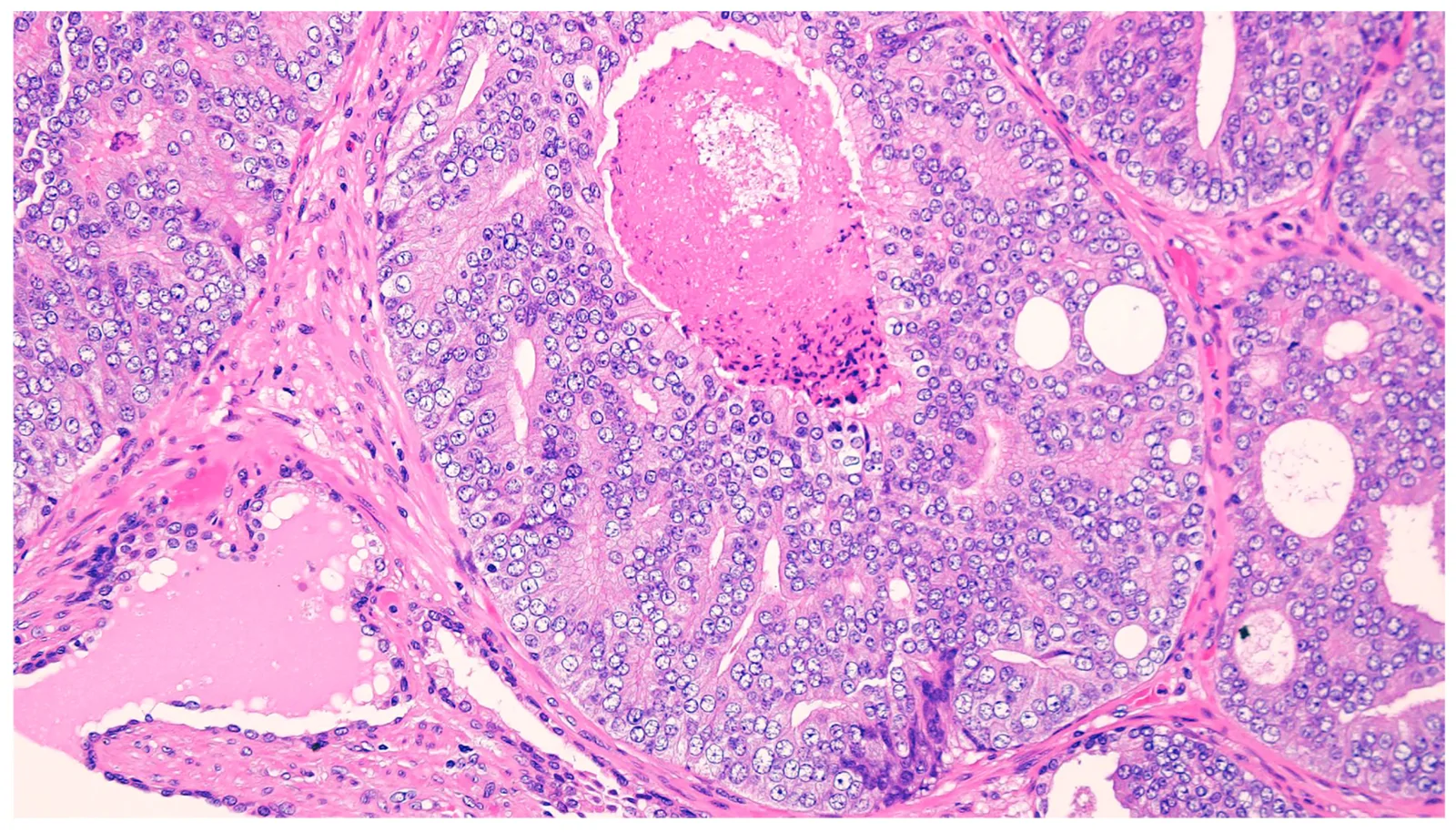
Intraductal carcinoma of the prostate. HE, ×200.

Intraductal carcinoma of the prostate (IDC-P) (Figure 11) was identified in 14 cases (20%).

**Figure 12:**
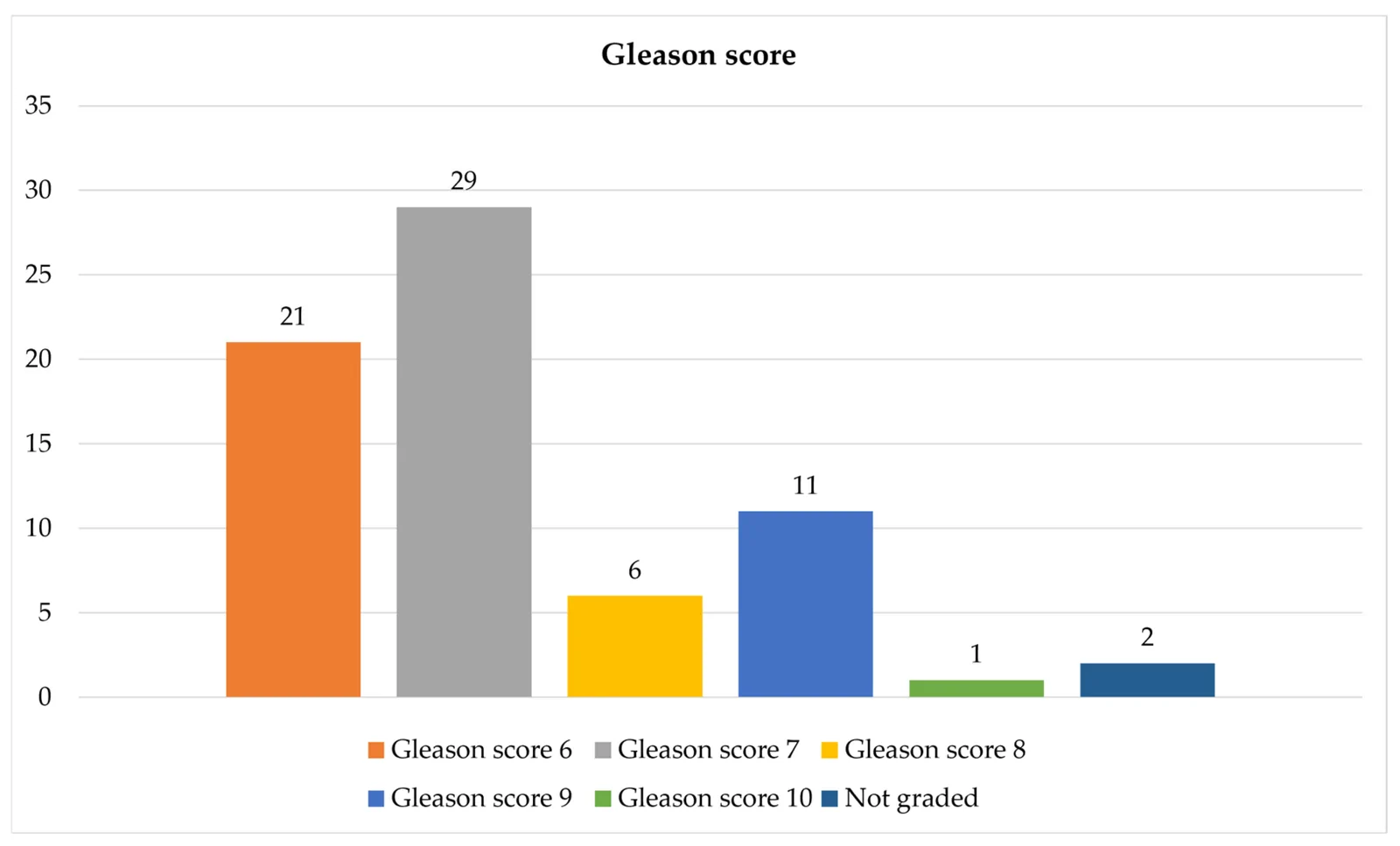
Distribution of cases according to Gleason score (n = 70).

The distribution of cases according to Gleason score (Figure 12) was as follows: GS 6: twenty-one cases (30%), GS 7: twenty-nine cases (41.43%), GS 8: six cases (8.57%), GS 9: eleven cases (15.71%), GS 10: one case (1.43%), ungraded: two cases (2.96%).

The bone and brain metastases from PC were not graded; however, the microscopic features were consistent with a poorly differentiated adenocarcinoma composed of cribriform aggregates, some with central necrosis, trabeculae, and solid nests of tumor cells.

The mean Gleason score was 7.15.

**Figure 13:**
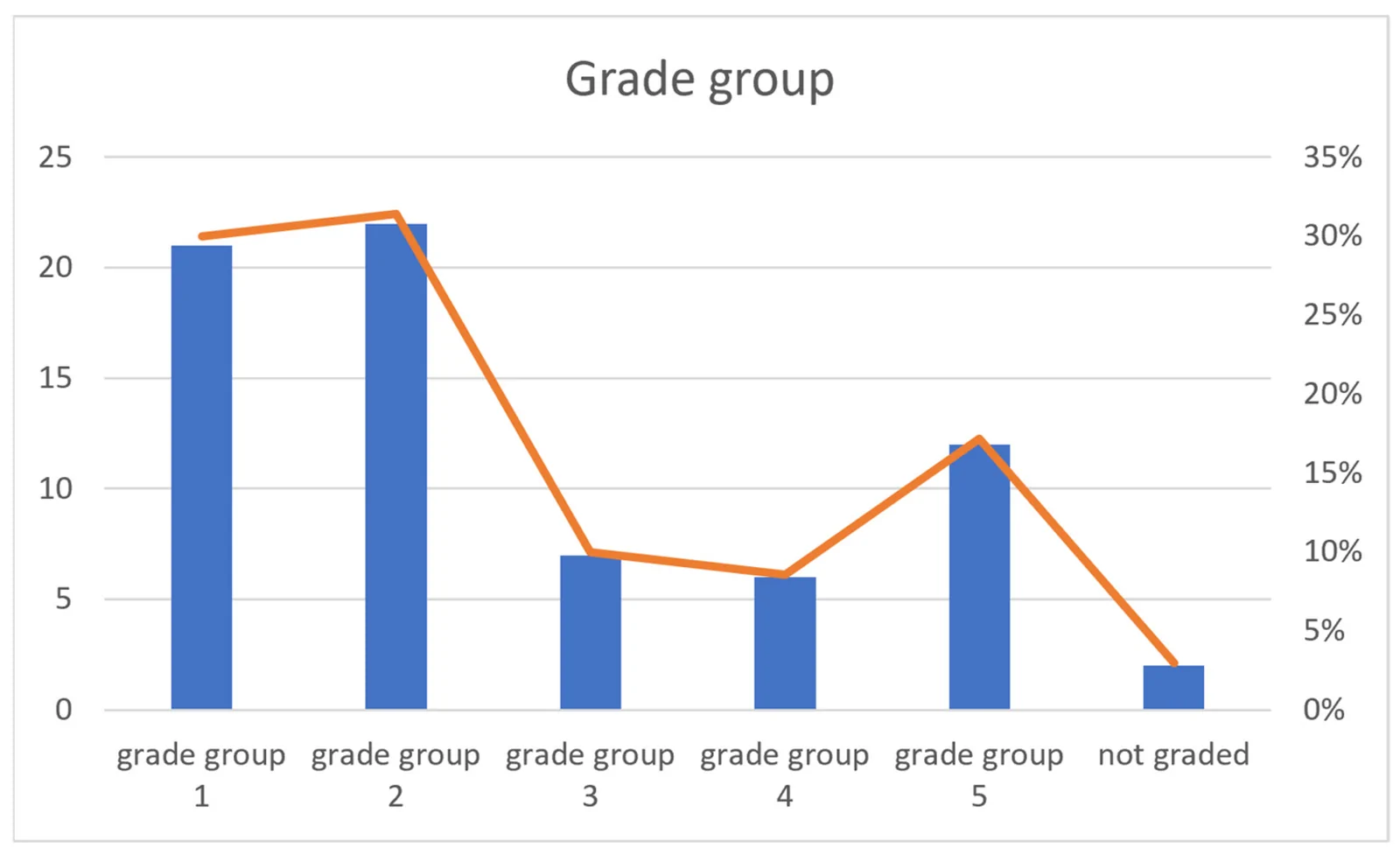
Distribution of cases according to prognostic grade group (n = 70).

The distribution of cases according to prognostic grade group was as follows (Figure 13): GG1: twenty-one cases (30%), GG2: twenty-two cases (31.43%), GG3: seven cases (10%), GG4: six cases (8.57%), GG5: twelve cases (17.14%), ungraded: two cases (2.96%). The mean prognostic grade group was 2.5. Grade group distribution did not differ significantly between young patients and the older (>55 years old) PC patients diagnosed during the study period, whether assessed by chi-square test (*p* = 0.394, Cramér’s V = 0.048) or by the Mann–Whitney U test with respect to the ordinal nature of the grade group (U = 53,948, *p* = 0.238, rank-biserial r = 0.082, 95% CI −0.06 to 0.22). An ordinal logistic regression yielded a common odds ratio of 0.76 (95% confidence interval 0.49–1.19, *p* = 0.225) for young patients having a higher grade group relative to the older patients, confirming the absence of a significant association.

### 3.9. Pathologic Stage and Other Microscopic Tumor Features

**Figure 14:**
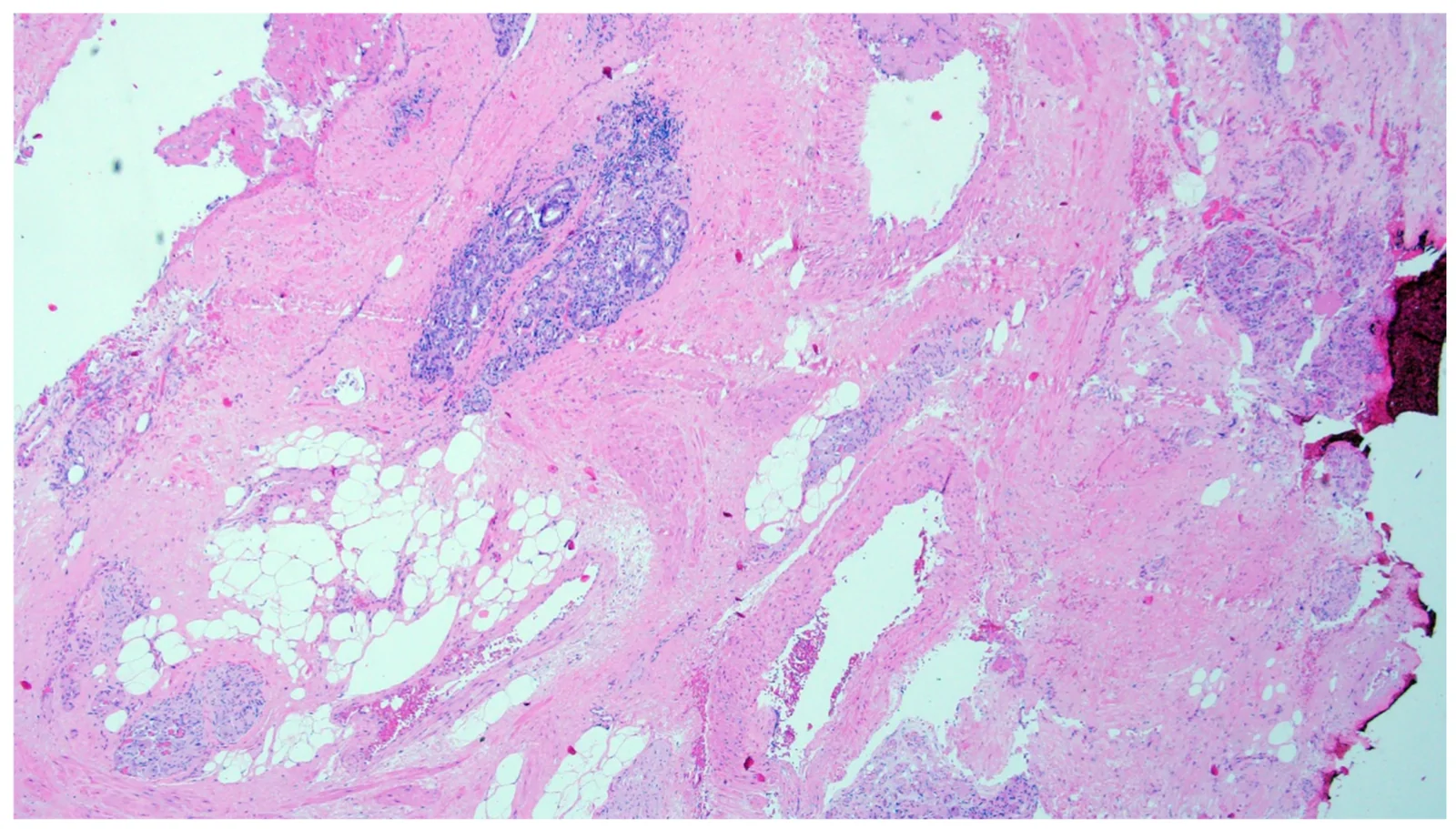
Extraprostatic extension. HE×40.

Among the 12 PC cases assessed on surgical excision specimens (RP and RCP), which allowed pathologic staging, seven tumors (58.33%) were organ-confined (pT2N0), whereas five (41.67%) had locally advanced disease (pT3N0/1/x) (Figure 14); two cases (16.67%) had positive resection margins (R1).

**Figure 15:**
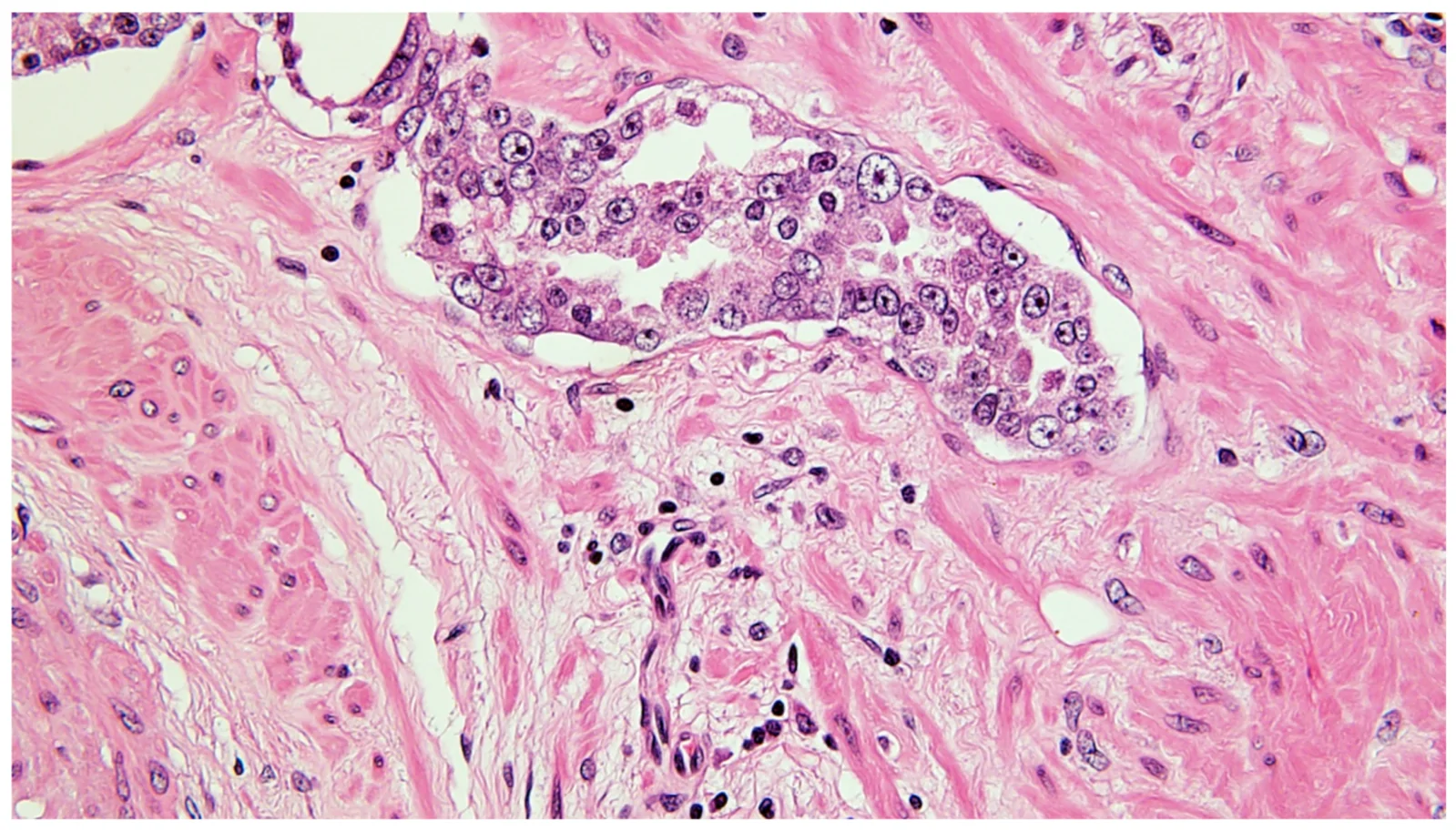
Invasion of lymphatic vessel. HE, ×200.

Perineural invasion (Pn1) was present in 37 (52.86%) of all cases in the cohort (n = 70), while lymphovascular invasion (Figure 15) was identified in 11 cases (15.71%) in the entire cohort.

### 3.10. Clinical Significance of Tumors

According to the criteria used, 49 of the 70 cases (70%) were considered clinically significant cancers. Only six of the seventy patients (8.57%) were eligible for active surveillance (AS)—localized tumors, PSA < 10, and Gleason score 6. A logistic regression was performed to test whether age and PSA level predicted clinically significant disease. Age was included because it defines the study cohort, while PSA was log-transformed [ln(PSA + 1)] due to its skewed distribution. Of the 70 young patients, 51 had complete data for both variables, of whom 37 (72.5%) had clinically significant disease. Higher PSA was independently associated with clinically significant disease: for each unit increase in ln(PSA + 1), the odds of clinically significant disease increased more than two-and-a-half-fold (OR 2.70, 95% CI 1.14–6.36, *p* = 0.023), while age was not a significant predictor (OR 1.02, 95% CI 0.74–1.41, *p* = 0.89).

## 4. Discussion

The introduction of PSA-based screening for PC detection has led to a decrease in the age at diagnosis of patients with this malignancy, from 72.2 years in the 1980s to 67.2 years in the 2000s [17], and to a nine-fold increase in incidence among men aged <55 years, as reported in the United Kingdom [4]. Our data regarding the proportion of PC in young men show substantial annual variation, with three peaks in 2023, 2021, and 2017. This fluctuation is difficult to explain, but the overall upward trend through 2024 and the peak reached in 2021, the year of the COVID-19 pandemic, are noteworthy and run counter to data from studies reporting reduced PC detection during that period due to decreased participation in screening programs [18] and reduced healthcare-seeking behavior among men with urologic complaints because of public fear, travel restrictions, and limitations affecting consultations and hospital admissions in that epidemiologic context. According to U.S. statistics, the incidence of PC in young men increased dramatically between 1975 and 2010, followed by a decline after 2010, although the incidence in 2015 remained six-fold higher than in 1975 [19]. Proposed contributors to the increasing incidence of PC in young men include improved detection, infections, familial and ethnic factors, environmental carcinogens, obesity, sedentary lifestyle, social determinants, and PSA-based screening [20]. The relationship with screening is difficult to sustain, however, since screening generally has not targeted individuals younger than 50 or 45 years, depending on eligibility criteria. Nevertheless, there are data indicating that despite recommendations from various professional bodies against routine screening, a small proportion of younger patients underwent PSA testing for reasons such as a family history of PC or specific clinical suspicion [19].

The proportion of 4% of PC in young men found in our study is close to the 5% reported in recent U.S. statistics for the same age group [21] but differs from older studies that reported an incidence/prevalence of around 10% in young patients [22], rising to as much as 34% in autopsy studies [23]. None of our patients were younger than 45 years, although the literature reports PC in men aged 30–39 years [8], and even younger, with 13 and 16 years being the youngest reported in the literature [19].

Comparison of incidence/prevalence data for PC in young men is difficult for several reasons. First, the definition of “young” varies across studies, with different cut-offs (between 50 and 55 years) separating younger from older patients [4], and some studies including [2] or excluding [8] patients aged 55 years. Second, variation in incidence may be explained by differences in cohort size, composition, and the method by which PC was diagnosed: during life [4,8] or postmortem [24,25].

Our data show a mean age at diagnosis of 70 years for all patients diagnosed with PC during the same time interval (2015–2024), which is extremely close to the mean age of 70–71 years reported in a U.S. study evaluating this parameter in patients diagnosed in the 1960s–1970s, prior to the implementation of PSA-based screening [17], a finding readily explained by the absence of such screening in our country.

Indeed, the availability of screening for PC remains intensely debated because, although it has reduced mortality by 25% in countries where it has been implemented, there are also data showing that it leads to overdiagnosis and overtreatment of indolent cancers that would not threaten the patient’s life, with sometimes undesirable treatment-related consequences such as urinary, sexual, and bowel dysfunction [26]. More recent guidelines recommend, on the one hand, shared decision-making regarding initiation of screening, and on the other hand, screening within the 45–70-year-old age range, with some variation in the proposed starting and stopping ages [27]. In this context, personalized screening for PC has been advocated, taking into account patient-specific factors that may allow a more accurate risk–benefit assessment [28].

The higher frequency of PC among men from urban areas (64.29%) observed in our study is consistent with the general tendency reported in international studies showing a predilection for prostate malignancy in this segment of the male population [29]. One such study reported a higher incidence in urban settings but higher mortality rates in rural areas, most likely related to more limited access to screening and specialist care allowing earlier diagnoses [30]. A study from North Carolina concluded that rural patients were less likely to undergo evaluation within multidisciplinary teams and less likely to be enrolled in active surveillance protocols for low-risk tumors, thereby being more often treated with RP or radiation therapy (RT) [31].

Prostate cancer in young patients is an important issue within male oncologic pathology, particularly when considering its socioeconomic implications, as these men are generally in the most active period of their lives.

The diagnosis of PC in young men may be challenging because clinicians may be reluctant to consider this possibility in younger men who sometimes present with atypical symptoms [32,33]. The slight upward trend in the frequency of PC in young men observed in our study, together with the predominance of cases diagnosed on CNB specimens, suggests increased referral of younger men to urologists and an appropriate diagnostic approach by the latter, namely the consideration of PC despite the relatively unusual age for this disease. The five cases in our series in which PC was identified in RCP specimens underscore the importance of measuring serum PSA even in young patients diagnosed with bladder tumors. These five cases of incidental PC (iPC) detected in RCP specimens displayed the characteristics of clinically insignificant PC, that is, cancers unlikely to threaten the patient’s life, which reinforces the notion that iPC is generally an indolent cancer unlikely to cause death [34]. Our cohort combines cases diagnosed across different specimen types, from CNB to RCP to metastatic biopsies, mirroring the actual case mix referred to our department rather than a preselected, uniform population. Given the limited sample size, the aggressiveness analysis was not stratified by specimen type, as doing so would leave each subgroup too small to interpret meaningfully; this heterogeneity should be kept in mind when reading our findings.

Data regarding tumor characteristics, natural disease course, and prognosis of PC in young patients remain controversial. Some studies suggest that PC in young men (≤55 years) may represent a distinct clinicopathologic subtype, with more rapid progression to disease-specific death than tumors occurring at older ages, especially in high-stage and high-grade disease [35]. In addition, early-onset PC has, according to some studies, a gene expression profile and inflammatory/immune pathway regulation that are different from PC arising in individuals older than 70 years [36]. On the other hand, other studies have shown that most young patients with PC have lower-stage and lower-grade tumors and better outcomes than those diagnosed at older ages [4,37], possibly related to fewer comorbidities, more aggressive treatment in younger men, and better treatment tolerance [2].

In addition to age at diagnosis, pre-treatment serum PSA, Gleason score/WHO-ISUP grade group, histologic subtype, tumor stage, and resection margin status are among the most important prognostic factors in PC [38], as they define disease aggressiveness. Given that a direct relationship has been reported between tumor stage, Gleason score, presence of metastases, and serum PSA level [39], and considering the median pre-treatment serum PSA value of 15 ng/mL in the analyzed cohort, it may be inferred that many PC cases in our study were advanced-stage tumors. Moreover, the mean Gleason score of 7.15 and mean WHO/ISUP grade group of 2.5, the high proportion of PC diagnosed on CNB specimens classified as intermediate- and high-risk (74.07%), as well as the high proportion of clinically significant cancers (70%) in our cohort, back up the view advanced by some researchers that PC in young patients is a more aggressive neoplasm [40]. This interpretation is further supported by the 20.29% of cases showing associated IDC-P, a proportion exceeding those reported in CNB and RP specimens (2.8% and 17.2%, respectively) for PC in general, without reference to a specific age group [41]. In fact, this proportion may be even higher, since although this entity was described more than 30 years ago [42], it was included in the WHO classification of prostate tumors only in 2016 [9]; therefore, the uropathologists involved in diagnosing PC at our institution could not have been expected to report it during the earlier years of the study period. Even after 2016, uropathologists encountered difficulties in diagnosing and reporting IDC because of unresolved or controversial issues concerning classification criteria and distinction from other invasive or intraductal lesions, such as invasive cribriform carcinoma, prostatic intraepithelial neoplasia (PIN), and atypical intraductal proliferations, as well as uncertainty about whether these lesions should be included in grading [43,44,45,46,47]. It is well-established that IDC, which represents colonization of ductal/acinar structures by invasive tumor cells and reflects an advanced stage of aggressive PC, is associated with unfavorable prognostic features such as high tumor volume, advanced disease stage (seminal vesicle invasion, increased likelihood of extraprostatic extension, and regional lymph node metastases), early biochemical recurrence, and shorter disease-free survival (DFS) [43,48,49]. The literature also indicates that patients with IDC more frequently harbor BRCA2 mutations, are younger at diagnosis, and have more biologically aggressive tumors [4]. Accordingly, NCCN guidelines recommend considering germline BRCA2 testing in young PC patients in whom IDC-P is identified in association with invasive adenocarcinoma [50].

Additional evidence supporting the aggressiveness of some PC cases diagnosed in young men in the present study includes the presence of ductal adenocarcinoma/adenocarcinoma with ductal features admixed with acinar adenocarcinoma in two cases (2.86%), as well as non-focal NE differentiation in two other cases (2.86%) of poorly differentiated adenocarcinoma (grade group 5). Ductal adenocarcinoma of the prostate is a rare histologic variant associated with lower serum PSA levels, more advanced stage at diagnosis, and high Gleason scores (8–10) [51], except for PIN-like ductal adenocarcinomas, which are assigned a Gleason score of 6 [52]. It also shows a tendency to metastasize not only to lymph nodes but also to less typical sites for PC, such as the lungs, brain, liver, penis, testis, adrenal gland, skin, and peritoneal cavity, and is associated with lower cancer-specific survival (CSS) compared with acinar adenocarcinoma [53]. There is no consensus on the optimal treatment for ductal adenocarcinoma of the prostate; however, given the tendency of some of these tumors to arise in the major prostatic ducts and the prostatic urethra, some authors consider cystoprostatectomy or radiotherapy more appropriate, due to the risk of peritoneal dissemination during RP [51].

The cribriform growth pattern of invasive carcinoma was identified in 23 out of 70 cases. The cribriform pattern of PC growth is found in acinar, ductal prostate adenocarcinoma or in IDC-P. The definition of cribriform carcinoma assumes the presence of confluent sheets of cohesive tumor cells that form secondary lumens visible at low magnification [54], with a sieve or Swiss-cheese appearance. Invasive cribriform carcinoma of the prostate must be differentiated from atypical intraductal proliferations (AIDP), from Gleason pattern 4 acinar carcinoma with fused glands or glomeruloid structures, from Gleason pattern 5 with pseudorosettes, from urothelial carcinoma involving the prostate ducts, from basal cell carcinoma of the prostate, or even from some benign lesions with a cribriform pattern (clear cell cribriform hyperplasia or cribriform basal cell hyperplasia) [55,56].

The cribriform pattern of acinar carcinoma, cribriform IDC-P, and cribriform ductal adenocarcinoma all have unfavorable prognostic significance and should therefore be mentioned in the pathology report as specified in the most recent ICCR reporting models for PC [57]. Furthermore, considering that ISUP and GUPS have reached a consensus on the inclusion of IDC-P in the grading of PC, provided that IDC-P is associated with invasive carcinoma and is not spatially distinct from it [58], discrimination of the two lesions no longer seems to be so important. Some studies emphasize the importance of the size of the cribriform epithelial aggregates, in the sense that large cribriform aggregates (those with >12 glandular lumens or with a diameter of more than 0.25mm) would be associated with an increased risk of metastasis [59], although other authors consider that both large cribriform and small cribriform glands have a similar adverse impact on patient outcomes [60].

The impact of NE differentiation on the course, prognosis, and treatment of PC remains intensively debated, beginning with the meaning of the term “NE differentiation”. Some authors use the term adenocarcinoma with diffuse NE differentiation, as found in the pathology reports of two patients in the present study, although this is not a category accepted in the current WHO classification of PC [61]. Diffuse NE differentiation may be documented both in de novo prostatic tumors and in hormonally treated tumors that undergo transdifferentiation, as part of so-called cellular plasticity, into castration-resistant NE carcinomas [62]. Although the prognostic significance of IHC-documented NE differentiation, reported in up to 100% of prostatic adenocarcinomas [63], in the absence of morphologic features of small-cell or large-cell NE carcinoma remains debatable, some authors consider prostatic adenocarcinomas with >50% of cells positive for NE markers (CgA, Syn) to be aggressive variants of prostatic adenocarcinoma [64]. From this perspective, it is highly likely that the two PC cases with diffuse NE differentiation in our series were aggressive tumors.

The presence of malignant tumors in young patients is a particularly relevant topic in the era of genetic testing, which increasingly encompasses PC and has implications for treatment and follow-up, and in some cases for family members as well. With regard to genetics in PC, the IMPACT study showed that among men carrying BRCA2 mutations, PC has a higher incidence than in non-carriers, occurs at younger ages, and many such cancers are clinically significant [65]. Although recent NCCN guidelines [66] recommend considering germline testing for susceptibility genes associated with PC—ATM, BRCA1, BRCA2, CHEK2, HOXB13, and TP53—in patients aged ≤55 years, our study lacks data on possible mutations in these genes because the high cost and the absence of functional infrastructure (equipment, trained staff, and experience) rendered such testing unavailable. In the absence of documented genetic findings in young patients diagnosed with PC, one possible recommendation would be to consider PSA-based screening for first-degree relatives beginning at 40–45 years of age [67].

Young age at diagnosis in men with PC may have therapeutic implications, insofar as the longer life expectancy of these patients, the risk of aggressive disease, and fertility- and quality-of-life-related issues all represent parameters relevant to treatment decision-making. In addition, young patients are more actively involved in the decision-making process and tend to seek information from multiple sources before choosing a treatment [68].

Treatment options for PC in young patients are multiple and depend on risk group, patient preference, associated conditions, available resources, and institutional expertise.

Follow-up of these patients should be rigorous and should include periodic PSA monitoring, imaging assessments, and clinical evaluation in order to detect recurrence, complications, or even a second malignancy at an early stage. A personalized follow-up protocol, taking into account risk factors and tumor grade, is recommended.

One strength of our study is the establishment of a patient database that may in the future allow the exploration of genetic mechanisms involved in prostate carcinogenesis, with potential implications for prevention, early diagnosis, and scientific research. Among the limitations of the study are the lack of complete data regarding family history and personal oncologic and therapeutic history, the small number of cases treated surgically at our institution, and the absence of follow-up data regarding the course of the disease. An additional limitation is that pathology diagnoses were not centrally reviewed again and were not retrospectively reclassified according to current WHO criteria; grading was based on the ISUP/WHO criteria in effect at the time of each original diagnosis. Since the study spans a 10-year period (2015–2024) during which diagnostic criteria evolved—most notably the formal inclusion of intraductal carcinoma of the prostate in the WHO classification only from 2016 onward—some inconsistency in case ascertainment and grading across the study period cannot be excluded.

## 5. Conclusions

Our findings indicate a low proportion of PC in younger patients; however, tumors in this population segment exhibit an aggressive clinico-morphological profile, which would warrant:

- Health education initiatives targeting the male population, focusing on the recognition of urinary symptoms and the importance of periodic clinical evaluation;
- Many younger patients showed unfavorable pathological features, supporting heightened vigilance and more complete risk documentation, with consideration for inclusion of PSA screening for patients aged 45 to 55 years, based on individual risk stratification (family history, race, ethnicity, genetic profile);
- Managing challenging cases within complex multidisciplinary teams, including urologists, oncologists, radiation oncologists, radiologists, geneticists, pathologists, and psychologists, to ensure accurate and timely diagnosis, and to establish individualized treatment and/or surveillance strategies, considering clinical, pathological, and genetic characteristics, as well as patient choices.

## Figures and Tables

**Table 1:** Comparison of demographic and pathological characteristics between prostate cancer patients aged ≤55 years and patients aged >55 years.

Characteristic	Patients ≤ 55 Years		Patients > 55 Years	
**Age (years)**	Range: 45–55 Median: 53 IQR: 2.5		Range: 56–93 Median: 70 IQR: 10	
**PSA (ng/mL)**	N = 51		N = 1300	
	**Raw PSA:** Range: 1.35–5182 Median: 15 IQR: 51	**ln(PSA + 1)** Range: 0.85–8.55 Median: 2.75 IQR: 1.94	**Raw PSA:** Range: 0.21–3801 Median: 12 IQR: 20	**ln(PSA + 1)** Range: 0.19–8.24 Median: 2.5 IQR: 1.2
**Gleason score** **(total)**	Range: 6–10 Mean: 7.15 Median: 7 Std dev: 1 IQR: 2		Range: 6–10 Mean: 7.20 Median: 7 Std dev: 1 IQR: 1	
**Grade Group**	Range: 1–5 Mean: 2.5 Median: 2 Std dev: 1.4 IQR: 3		Range: 1–5 Mean: 2.64 Median: 2 Std dev: 1.4 IQR: 2	

PSA: prostate-specific antigen; IQR: interquartile range; ln(PSA + 1): natural logarithm of PSA + 1, used to correct for the right-skewed distribution of PSA values; Std dev: standard deviation.

**Table 2:** Clinicopathological characteristics of the young patient cohort (aged ≤55 years, n = 70).

Characteristic	Patients ≤ 55 Years (n = 70)	
**Age (years)**	Range: 45–55 Median: 53 IQR: 2.5	
**PSA (ng/mL)**	**Raw PSA** Range: 1.35–5182 Median: 15 IQR: 51	**ln(PSA + 1)** Range: 0.85–8.55 Median: 2.75 IQR: 1.94
**Specimen type, n (%)**	CNB: 54 (77%) TURP: 7 (10%) RP: 2 (3%) RCP: 5 (7%) Biopsy from metastasis: 2 (3%)	
**Grade Group, n (%)**	Grade group 1: 21 (30%) Grade group 2: 22 (31%) Grade group 3: 7 (10%) Grade group 4: 6 (9%) Grade group 5: 12 (17%) Not graded: 2 (3%)	
Risk group *, n (%)	Low risk: 4 Intermediate risk: 20 High risk: 19 Undeterminable risk: 6	
**IDC-P, n (%)**	14 cases (20%)	
**Invasive cribriform carcinoma, n (%)**	24 cases (34%)	
**Lymphovascular invasion, n (%)**	11 cases (15%)	
**Perineural invasion, n (%)**	37 cases (53%)	
**Clinically significant disease, n (%)**	49 cases (70%)	

PSA: prostate-specific antigen; IQR: interquartile range; ln(PSA + 1): natural logarithm of PSA + 1; CNB: core needle biopsy; TURP: transurethral resection of the prostate; RP: radical prostatectomy; RCP: radical cystoprostatectomy; IDC-P: intraductal carcinoma of the prostate. * Risk group was assigned according to European Society for Medical Oncology (ESMO) criteria and could not be determined in cases with unavailable PSA values.

## Data Availability

The data supporting the findings of this study are available from the corresponding author upon reasonable request.

## Abbreviations

The following abbreviations are used in this manuscript:

ADT	Androgen deprivation therapy
AJCC	American Joint Committee on Cancer
AS	Active surveillance
CNB	Core needle biopsy
CT	Computed tomography
DAB	3,3’-diaminobenzidine
DFS	Disease-free survival
ESMO	European Society for Medical Oncology
GUPS	Genitourinary Pathology Society
HE	Hematoxylin and Eosin
IDC-P	Intraductal carcinoma of the prostate
iPC	Incidental prostate cancer
ISUP	International Society of Urological Pathology
LVI	Lymphovascular invasion
PBCECHT	“Pius Brînzeu” County Emergency Clinical Hospital in Timișoara
PC	Prostate cancer
PET/CT	Positron Emission Tomography-Computed Tomography
PIN	Prostatic intraepithelial neoplasia
PNI	Perineural invasion
PSA	Prostate-Specific Antigen
NCCN	National Comprehensive Cancer Network
NE	Neuroendocrine
RP	Radical prostatectomy
RT	Radiotherapy
TNM	Tumor, Node, and Metastasis cancer staging system
WHO	World Health Organization
